# Editorial: Novel and emerging therapies for the treatment of obesity and related disorders

**DOI:** 10.3389/fendo.2024.1371113

**Published:** 2024-04-26

**Authors:** Isabel R. Amado, Marina Romaní-Pérez, Pablo Fuciños, Manuel Gil-Lozano

**Affiliations:** ^1^ International Iberian Nanotechnology Laboratory (INL), Braga, Portugal; ^2^ Microbiome, Nutrition & Health Research Unit, Institute of Agrochemistry and Food Technology, Severo Ochoa Centre of Excellence, National Research Council (IATA-CSIC), Valencia, Spain; ^3^ Institute for Diabetes and Cancer, Helmholtz Munich, Neuherberg, Germany; ^4^ Joint Heidelberg-Institute for Diabetes and Cancer (IDC) Translational Diabetes Program, Inner Medicine, Heidelberg University Hospital, Heidelberg, Germany; ^5^ German Center for Diabetes Research (DZD), Neuherberg, Germany

**Keywords:** obesity, body composition, insulin resistance, microbiota, intestinal inflammation, exercise mimetics, brown adipose tissue (BAT), extracellular vesicles

Obesity and its associated comorbidities represent a significant burden for healthcare systems worldwide. The current Research Topic includes studies dealing with various topics relevant to obesity, from novel and revolutionary therapies to the diagnosis and physiological characterization of the disease; which contribute to improve our understanding of this important medical challenge.

## Assessment of body composition in obesity

Obesity is defined by excessive adipose tissue mass, and people are classified as obese when their body mass index (BMI) is over 30 kg/m^2^. However, using BMI as a diagnostic criterion has limitations, as it does not provide information about the subjects’ body composition. Computed tomography (CT) at the lumbar area can assess body composition with great accuracy; however, it is an expensive and time-consuming process that requires highly trained individuals. Dual-energy X-ray absorptiometry is less laborious but tends to underestimate fat mass in obese subjects. Palmas et al. eveloped a model able to analyze CT images in an automated way, based on artificial intelligence. Although similar approaches have been utilized to evaluate body composition in cancer patients, it is the first time a tool of this kind was designed for obese subjects as the target population. As a result, the new model was more precise at assessing fat and fat-free mass in obese individuals. These findings will simplify the analysis of body composition in obesity, while still maintaining the inherent advantages of CT, including accurate evaluation of visceral and subcutaneous fat as well as sarcopenia.

## Obesity and insulin resistance

Obesity results from an imbalance between energy consumption and expenditure, and it is commonly associated with increased insulin resistance. However, how insulin resistance develops in the context of obesity remains to be clarified. Cooper et al. evaluated glucose fluctuations in obese subjects with normoglycemia by continuous glucose monitoring (CGM). Despite the consumption of a high carbohydrate diet, the daily glucose profile of those individuals was comparable to healthy controls. However, the obese subjects showed significantly higher insulin levels and HOMA-IR; suggesting that enhanced insulin release might be secondary to alterations induced by excess adiposity rather than a direct consequence of acute changes in glycemia, although the potential role of incretins could not be discounted. Moreover, the study indicates that fasting insulin and HOMA-IR values are more valuable than CGM in managing obesity from a clinical perspective.

## Other pathologic consequences of obesity

Nonalcoholic fatty liver disease (NAFLD) represents one of the most frequent comorbidities associated with obesity. Jiang et al. explored the role of extracellular vesicles (EVs), released by different tissues, as signaling factors in NAFLD; revealing their significant impact on the pathology of the disease. The authors found that EV abundance and content change dynamically through the different stages of NAFLD, indicating their potential as stage-specific markers. The study also highlights EVs as promising drug delivery systems with low immunogenicity and high biocompatibility. Overall, it suggests that research on EVs in NAFLD shows potential for future diagnostic and therapeutic applications. Obesity also increases the risk of venous thromboembolism (VTE). Low-molecular-weight heparin (LMWH) is commonly used for the treatment of VTE. However, LMWH dosage is the same across different weight groups, including obese subjects, although a low distribution into adipose tissue and total body clearance has been reported in these patients as compared to normal-weight individuals. Liu et al. conducted a systematic review and meta-analysis to explore the appropriate dosage of LMWH for preventing and treating VTE in patients with obesity. They concluded that a higher dosage of LMWH in patients with obesity can reduce the incidence of VTE without increasing the risk of bleeding.

## Life-style intervention mimics in the treatment of obesity

Life-style interventions are the first line of therapy for the treatment of obesity; however, this approach often fails to maintain health benefits in the long-term due to low adherence. Herein, Yi et al. reviewed the current clinical and preclinical data on the multiple metabolic benefits promoted by β-aminoisobutyric acid (BAIBA), a novel myokine released by skeletal muscle in response to physical activity. Such benefits include stimulating glucose uptake by peripheral tissues, improving glucose homeostasis, regulating hepatic lipid metabolism, reducing steatosis, and increasing browning and diminishing white adipose tissue depots, all of them highly desirable in the context of obesity. Accordingly, BAIBA is presented as an exercise mimic that could be used as therapy in obese subjects who cannot stick to a consistent exercise regime.

## Intestinal inflammation, gut microbiota and obesity

Bariatric surgery can achieve remarkable weight loss. However, it is a highly invasive intervention only recommended for severely obese patients who fail to lose weight after trying other forms of therapy. Active research is going on to elucidate how bariatric surgery elicits its metabolic benefits. In this regard, Cao et al. investigated the effects of sleeve gastrectomy (SG) on colonic inflammation in a model of diet-induced obesity. The authors described substantial reductions in inflammatory markers and increments in markers of intestinal barrier integrity in response to SG.

Moreover, they demonstrated that depletion of gut microbiota highly attenuated these changes, emphasizing the relevant role of the microbiome in the metabolic consequences of bariatric surgery. In addition, Wang et al. studied the hypoglycemic, antioxidant, and anti-inflammatory activity of polysaccharides from the fungus *Cordyceps cicadae* in diabetic mice, also focusing on their effect on the intestinal microbiota. Mice treated with these polysaccharides showed reduced blood glucose levels and antioxidant and anti-inflammatory responses, as confirmed by alleviated tissue damage compared to the control. The microbiota of treated mice had reduced Firmicutes/Bacteroidetes ratio and significantly decreased *Helicobacter* and *Lactobacillus* compared to the diabetic group. Interestingly, the hypoglycemic effects of these polysaccharides were linked to metabolites produced by certain microbiota species increased in the gut of the treated group, highlighting the interest in these natural compounds for treating diabetes. Related to this, Zhang et al. reviewed the evidence linking intestinal barrier dysfunction with the development of systemic inflammation and metabolic alterations. The authors summarized the different strategies available to improve the integrity of the intestinal barrier and how these interventions may affect obesity and other metabolic diseases. Overall, these three studies highlight the importance of preserving intestinal barrier integrity and reducing intestinal inflammation in the context of obesity. Although certain drugs and natural compounds such as metformin, berberine and butyrate have shown promising effects in preclinical studies, more clinical investigation is needed. Importantly, interventions to regulate the composition of intestinal flora, such as oral supplementation with prebiotics, probiotics and postbiotics are currently under consideration for the treatment of obesity.

## Brown adipose tissue as target for the treatment of obesity

Since its identification in humans, activation of BAT and induction of thermogenesis have received much attention as potential strategies for maintaining body weight and improving the metabolic alterations often associated with obesity. However, it remains controversial whether activation of the limited amount of BAT in humans could be sufficient to trigger metabolic effects. Zhu et al. explored the provocative idea of BAT or engineered thermogenic cell transplantation to improve metabolic abnormalities. The authors reviewed the preclinical data on brown fat transplants and the *in vitro* generation and subsequent implantation of thermogenic cells. They also assessed the current severe limitations of this technique that preclude the adoption of such intervention at this point, including the progressive loss of the thermogenic phenotype in the implanted cells. More research on this area is warranted to improve the viability of this strategy, while some ideas on how to advance in this direction are already presented in the manuscript.

## Conclusion

Historically, pharmacotherapy of obesity has enjoyed limited success because of modest weight-loss effects as well as important side effects such as addiction, cardiovascular events and gastrointestinal complications. Remarkably, novel and revolutionary therapies based on GLP-1 derivative drugs can induce body weight reduction by up to 24% (1), highlighting the importance of innovation for the generation of effective anti-obesity medications. Despite the dramatic effects of these drugs, they are not well-tolerated by a considerable fraction of the patients, and discontinuation of the treatment is associated with a remarkable rebound in body weight. Thus, the search for novel medications is still highly relevant. This Research Topic covers a wide range of potential anti-obesity therapies including exercise mimics, BAT transplantation, microbiota-based strategies, drugs to reduce intestinal inflammation as well as the use of extracellular vesicles as delivery method.

## Author contributions

IA: Writing – original draft, Writing – review & editing. MP: Writing – original draft, Writing – review & editing. PF: Writing – original draft, Writing – review & editing. MG: Writing – original draft, Writing – review & editing.
